# Supplementary figures and images for: Sialokinin in mosquito saliva shifts human immune responses towards intracellular pathogens
Source: PLoS Negl Trop Dis. 2023 Feb 3;17(2):e0011095. doi: 10.1371/journal.pntd.0011095 (PMC9897557; doi:10.1371/journal.pntd.0011095)

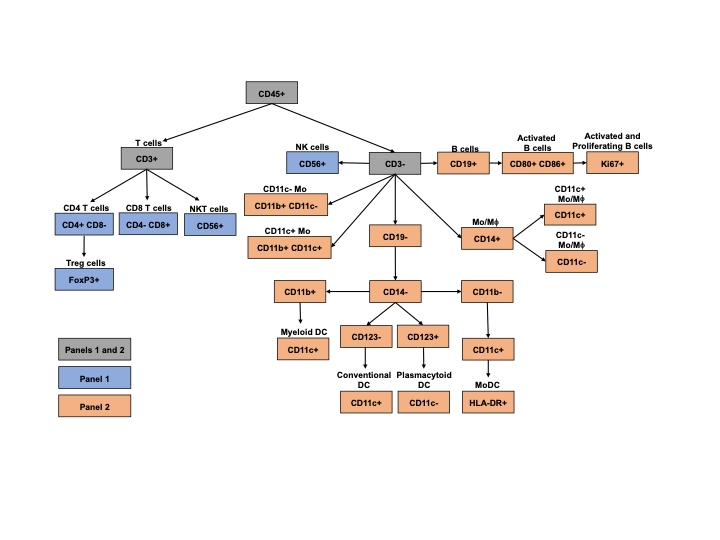

Supplement: S1 Fig — (TIFF) [file pntd.0011095.s001.tiff]
