# Supplementary material for: Sialokinin in mosquito saliva shifts human immune responses towards intracellular pathogens
Source: PLoS Negl Trop Dis. 2023 Feb 3;17(2):e0011095. doi: 10.1371/journal.pntd.0011095 (PMC9897557; doi:10.1371/journal.pntd.0011095)
Supplement: Attachment — Submitted filename: ResponsetoReviewers_Final.docx [file pntd.0011095.s002.docx]

Responses to Reviewers

We thank the reviewers for their thorough review of our manuscript and insightful commentary. Their insights will undoubtedly make our manuscript better. Responses to individual comments are described below in red. We have noted where specific changes were made in the manuscript by providing line numbers where applicable.

Reviewer #1: This is a well-conducted and nicely written study. The objective was to determine the effect of sialokinin (a potential immunomodulatory mosquito saliva protein) on the human immune response through the use of humanized mice. The mice were fed on by wildtype mosquitoes or sialokinin knockout mosquitoes (two strains were used) or they received sialokinin via needle inoculation. All of my concerns are extremely minor.

Line 77: “or bitten by uninfected mosquitoes” – surely this is a mistake because disease would not be expected in mice bitten by these mosquitoes
We have made the appropriate changes. Please see line number 77 in the updated manuscript.

Line 89: replace “include” with “are the”

We have made the appropriate change. Please see line number 89 in the updated manuscript.

Line 213: considering mentioning the % of FBS the first time “PBS/FBS” is used

We have made the suggested change. Please see line number 224 in the updated manuscript.

Line 228: replace Table with Table 2. Same applies to line 231.

The text described on lines 228 (new version line 239) and 231 (new version line 242) state Table 2. No changes were made.

Lines 253-254: I can see the CD11c+ data in Figure 1, but not the data for CD45+, CD3- and CD14+. Consider mentioning “data not shown” where applicable. Same applies to later figures.

The notations listed for CD45+, CD3-, and CD14+ are the markers used to identify total monocyte and macrophage populations by flow cytometry. All monocytes or macrophages are further broken down into CD11c+ or CD11c- subsets of only monocytes, or both monocytes and macrophages. We have altered the text to clarify the markers used for total monocyte and macrophage populations. Please see lines 266-267.

Reviewer #2: The manuscript “Sialokinin in mosquito saliva shifts human immune responses towards intracellular pathogens” explores the potential immunomodulatory activity of sialokinin using humanized mice, which is of great importance to the field. The manuscript is well ordered and easy to understand. Overall, my main concern is the significant difference in results that can be observed between the two KO strains. Additionally, the injection of sialokinin, in most cases, does not “recover” the observed phenotype. Therefore, is not clear to me how the authors reached some of their conclusions. Below I present a few specific questions to the authors.

1.      In lines 200 – 201, the authors state that 18 ug of sialokinin were injected in each animal. What was the rationale for this concentration? Did the authors perform a pilot experiment using different concentrations of sialokinin? Considering that mosquito salivary glands contain 1 – 2 ug of total protein and it is speculated that not all salivary content is injected during the feeding cycle, it is more likely that only a few nano-grams of sialokinin are injected into the host.

We thank the reviewer for this relevant point. The amount of sialokinin initially indicated in the methods (180ug/mL) was the concentration of stock solution that was further diluted prior to injection. For this study, mice received 3600ng of sialokinin peptide per injection. We have altered the text to reflect the correct concentration of sialokinin injected per mouse on lines 202-203. The concentration of sialokinin per injection was chosen based on the amount of sialokinin present in salivary glands. Studies have shown that there is roughly 700ng of sialokinin I in a pair of *Ae. aegypti* salivary glands (Champagne and Ribeiro, 1994). Since bitten mice received four mosquito bites by wild-type or knockout mosquitoes for the comparative studies, we multiplied the concentration of sialokinin by four and added approximately 20% to account for discrepancies in pipetting, mixing, and injection. We have included this rationale for the concentration of sialokinin protein used on lines 204-209.

2.      Most results showed that the injection of sialokinin did not “recover” the observed phenotype when wild-type mosquitoes were used. One example of this is provided in figure 1C, in which an increase of CD11c- monocytes and macrophages were found when comparing KO and wild-type groups. However, the same effect is found in the groups injected with sialokinin or saline. If the increase of CD11c- population is due to the lack of sialokinin in the mosquito saliva, how did the injection of sialokinin yield the same result?

While injection with sialokinin was hypothesized to rescue phenotypes affected by sialokinin knockout mosquito bites, several cell populations did not show such an effect. While this is an interesting and unexpected result, it is important to consider the interplay between mosquito salivary proteins within the host. With this in mind, it is possible that the differences in “recovery” responses were due to injection of the sialokinin peptide alone and not the full breadth of mosquito salivary proteins present in the knockout bites. The lack of additional salivary proteins in the sialokinin injected groups could be playing a role. We have elaborated on this point in the discussion section on lines 452-458.

3.      Some of the results presented in the current manuscript show inconsistencies between the two KO strains. One example is in lines 287 – 289, in which the authors state that plasmacytoid dendritic cells were higher in the bone marrow of mice bitten by sialokinin d8 KO, but an opposite effect was observed for the d5 KO (Figure 2D). These “contradictory” results can also be observed in other panels of figures 2, 3 and 4. Could the authors provide an explanation or a comment on this? Could the KO of sialokinin result in additional changes in the composition of mosquito saliva?

We thank the reviewer for this important point. The differences in immune cell populations of mice bitten by the d5 or d8 knockout mosquitoes could be due to differences in generating the knockout lines. The mosquito knockouts were generated by CRISPR/Cas9 technology using two different guide RNAs to allow for comparisons of off-target effects. CRISPR/Cas9 deletions could have yielded additional mutations in other genes, causing a change in mosquito saliva composition. Future studies will be needed to further characterize total mosquito saliva composition between the two knockout lines.

We have elaborated on this point on lines 440-451 in the discussion section.

4.      Data for the sialokinin- and saline- injected groups are missing in figure 4.

We thank the reviewer for pointing out this oversight. Those data have been added to Figure 4.

Reviewer #3: In the current study, Clinton et al., studied the role of Sialokinin; a mosquito saliva protein, in immune responses regulations following the mosquito bite. Although the Sialokinin has already been studied in details however majority of these studies are performed on wild-type C57BL/6 and BALB/c mice hence there is a need for studies which can insight more about molecular cross talk and immune responses. In the current study, authors used humanized mice and studied immune cells regulation following the bite with sialokinin knockout or wild type Ae aegypti. Overall this is nice study however I have few concern authors need to address-

1.      Authors need to validate if mosquitoes are sialokinin knockout using western blotting.

These knockout mosquito lines were generated by another research group and validated extensively in their publication. The citation Martin-Martin et al., 2022 is referenced on lines 163 and 168 when describing the knockout mosquito generation. We also describe their methods of validation on lines 166-168.

2.      Authors also need to check if addition of mosquito saliva or salivary gland extract alone to human immune cells like THP1 can alter the immune responses.

Although these experiments would be interesting to perform, we have performed studies in cell types that are considered more relevant to the human immune system. For example, we previously published studies regarding altered immune responses by treating immune cells isolated from human donors (PBMCs) with mosquito saliva. This study, by Vogt et el., 2018, was cited in this manuscript on line 87. We have elaborated on this point on lines 86-87.

3.      Please incorporate FACS graphs to the result section.

For clarity of the results section, we have chosen to include a representative figure depicting the gating strategy of markers used for flow cytometric analysis. Including all flow cytometry plots as figure components would be too unwieldy to include in the results section. In lieu of including all flow cytometry plots, we will be providing access to all raw flow cytometric data per standard data sharing and publication agreements. Please see Supplementary File Figure 1 for the representative flow cytometric gating strategy plots. We have described the gating strategy figure on lines 245-247.
